# Biocompatibility of Bacterial Magnetosomes as MRI Contrast Agent: A Long-Term In Vivo Follow-Up Study

**DOI:** 10.3390/nano11051235

**Published:** 2021-05-07

**Authors:** Xiaohui Nan, Wenjia Lai, Dan Li, Jiesheng Tian, Zhiyuan Hu, Qiaojun Fang

**Affiliations:** 1 Laboratory of Theoretical and Computational Nanoscience, CAS Key Laboratory of Nanophotonic Materials and Devices, CAS Key Laboratory of Standardization and Measurement for Nanotechnology, CAS Key Laboratory for Biomedical Effects of Nanomaterials & Nanosafety, CAS Center for Excellence in Nanoscience, Beijing Key Laboratory of Ambient Particles Health Effects and Prevention Techniques, National Center for Nanoscience and Technology, Chinese Academy of Sciences, Beijing 100190, China; nanxh@nanoctr.cn (X.N.); laiwj@nanoctr.cn (W.L.); lid@nanoctr.cn (D.L.); 2 University of Chinese Academy of Sciences, Beijing 100049, China; 3 State Key Laboratories for Agrobiotechnology, College of Biological Sciences, China Agricultural University, Beijing 100193, China; tianhome@cau.edu.cn; 4 Sino-Danish Center for Education and Research, University of Chinese Academy of Sciences, Beijing 100190, China

**Keywords:** bacterial magnetosomes (BMs), clearance, biocompatibility, targeted organs, magnetic resonance imaging (MRI)

## Abstract

Derived from magnetotactic bacteria (MTB), magnetosomes consist of magnetite crystals enclosed within a lipid bilayer membrane and are known to possess advantages over artificially synthesized nanoparticles because of the narrow size distribution, uniform morphology, high purity and crystallinity, single magnetic domain, good biocompatibility, and easy surface modification. These unique properties have increasingly attracted researchers to apply bacterial magnetosomes (BMs) in the fields of biology and medicine as MRI imaging contrast agents. Due to the concern of biosafety, a long-term follow-up of the distribution and clearance of BMs after entering the body is necessary. In this study, we tracked changes of BMs in major organs of mice up to 135 days after intravenous injection using a combination of several techniques. We not only confirmed the liver as the well-known targeted organs of BMs, but also found that BMs accumulated in the spleen. Besides, two major elimination paths, as well as the approximate length of time for BMs to be cleared from the mice, were revealed. Together, the results not only confirm that BMs have high biocompatibility, but also provide a long-term in-vivo assessment which may further help to forward the clinical applications of BMs as an MRI contrast agent.

## 1. Introduction

Bacterial magnetosomes (BMs) are natural magnetic nanomaterials synthesized by magnetotactic bacteria (MTB), consisting of magnetite crystals enclosed within a lipid bilayer membrane [1,2,3]. The core mineral part of the magnetosome is mainly composed of either magnetite (Fe_3_O_4_) or greigite (Fe_3_S_4_), while the peripheral membrane is highly similar to the cell membrane [4,5,6,7,8]. The biogenic membranes not only make BMs stable and disperse well, but also provide a large number of functional groups and proteins to allow easy isolation, purification, and modifications [9,10]. BMs display narrow size distribution and uniform morphologies in a single magnetotactic bacterial species or strain [11]. The process of the biomineralization of iron crystals and the formation of BMs are subject to strict gene control which guarantees good crystallinity and uniformity of the particle size and morphologies [11,12,13]. It is noteworthy that among various MTBs, *Magnetospirillum gryphiswaldense* MSR-1 is known as the most suitable MTB strain for medical applications, due to the fast growth rate and high productivity at >10 mg of BMs per liter, as well as the non-toxic culturing condition [14]. 

Studies on the applications of BMs are increasing in recent years, especially in the biomedical field. As superparamagnetic nanoparticles, BMs were reported to have higher transverse relaxivity (r2) and more effective signal decay than chemically synthesized nanoparticles as contrast agents for magnetic resonance imaging (MRI) application [15,16,17]. Several studies have also shown that BMs possess great potential to be used as an MRI contrast agent after modification to actively target tumors due to sufficient spatial resolution, together with high sensitivity and biocompatibility, as well as low dosage in preclinical studies [18,19,20,21,22,23]. BMs were reported as magnetic resonance imaging (MRI) contrast agents for molecular imaging of brain tumors and can be modified with peptides for targeted MRI to acquire improved transverse relaxation decay rate (R2) [17,24]. Mériaux et al. examined the MRI contrasting efficiency of BMs in mouse brains and found that a low dose of BMs as few as picomoles per kilogram can still be detected with an ultra-high field MRI scanner [19]. Besides, applications of BMs in enzyme immobilization, food safety, cell separation, and cancer therapy using magnetic hyperthermia or as drug delivery carriers have drawn great attention as well [25,26,27,28,29,30]. 

As an MRI contrast agent, applications of BMs in vivo require systematic studies on the toxicity and biocompatibility, including their distributions and clearance after intravenous injection. However, so far, most researches focus on the potential applications of BMs, and few have reported their toxicity and biosafety. Sun et al. examined for the first time the systematic effect of BMs on rats for the acute toxicity, immunotoxicity, and cytotoxicity of BMs from MSR-1 [22]. However, this work only followed up to two weeks and did not monitor the distribution changes of BMs in vivo during the two weeks. As the core of BMs is inorganic magnetite which cannot be easily degraded, it is necessary to conduct a long-term in-vivo study to track their dynamic distributions in various organs and understand the elimination pathway in mice. 

In this study, we have performed long-term in-vivo and in-vitro experiments to track the distribution and clearance of BMs. We monitored BMs after intravenous injection for 135 days by magnetic resonance imaging (MRI) of mice and found that BMs could cause fast signal decay in T2-weighted images of the liver and spleen, and the signal was gradually recovered with the clearance of BMs, indicating that the targeted organs were liver and spleen. Prussian blue staining and Haematoxylin and Eosin (HE) staining of tissues further verified MRI results and further showed good biocompatibility of BMs. Moreover, inductively coupled plasma mass spectrometry (ICP-MS) was applied to quantitatively analyze the iron content at different time points after intravenous injection. Together with the degradation of BMs in cells observed by immunocytofluorescence (ICF) imaging, a picture of the distribution and elimination paths of BMs in mice was obtained. We expect that this work will help to understand the biocompatibility and in-vivo behavior of BMs, and can provide a reference for the future clinical applications of BMs.

## 2. Materials and Methods

### 2.1. Preparation of Bacterial Magnetosomes (BMs)

*Magnetospirillum gryphisiwaldense* MSR-1 (DSM 6361, Brunswick, Germany) was obtained from China Agricultural University. The wild-type magnetotactic bacteria MSR-1 were cultured at 30 °C and 100 rpm/min in the medium as described in our previous study [24]. The bacteria were collected by centrifugation at 12,000 rpm for 5 min. To extract the BMs, MSR-1 cells were re-suspended in 10 mL phosphate-buffered solution (PBS 10 mM, pH 7.4) and sonicated for 26 min at 200 W (working 3 s, intermittent 5 s) [24,31,32]. After bacterial lysis, BMs were separated from the bacterial debris using a magnet overnight. Afterwards, the supernatant was discarded, the absorbed BMs were resuspended by PBS to continue sonicating for 26 min at 120 W and then positioned against a magnet for 4 h to attract the BMs. This procedure was repeated twice with the power changing from 80 W to 40 W, respectively. After ultrasonic, the BMs were washed twice with ultrapure water, then lyophilized into powder and stored at −20 °C for later use.

### 2.2. Preparation of the Polyclonal Antibody of BMs

Two healthy New Zealand rabbits were used to prepare polyclonal antibodies against the surface proteins of BMs, which were then used for the localization of BMs in subsequent Immunocytofluorescence (ICF) assay. The rabbits were immunized with 1 mL BMs (2 mg/mL) that were fully emulsified with the same dose of Freund’s complete adjuvant and injected into the back of rabbits at multiple points for the first immunization. Then 1 mL BMs (0.5 mg/mL) were fully emulsified with the same dose of incomplete Freund’s adjuvant to enhance the immunization every 2 to 3 weeks. Afterwards, the serum of rabbits was taken for Elisa detection after three immunizations. If the antibody titer was above 1:16,000, all serum would be collected for purification. If the antibody titer was lower than 1:16,000, the immunization strengthening step was continued until qualified. Finally, all serum of each rabbit was taken and purified by protein affinity column chromatography.

### 2.3. Transmission Electron Microscopy (TEM) Analysis

TEM (JEM-1230, JEOL, Tokyo, Japan) was used to visualize the morphological characteristic of BMs. BMs were washed three times with ultrapure water and resuspended in ultrapure water to make a stock solution, then diluted to an appropriate concentration with ultrapure water. The sample was dropped on a duplex copper grid coated with carbon film to incubate for 3 min and air-dried at room temperature. Images were recorded at an accelerating voltage of 80 kV.

### 2.4. Size and Zeta Potential Measurement

Zetasizer Nano ZS (Malvern Instruments Ltd., Worcestershire, UK) was used to determine the surface charge and the size distribution of BMs dispersed in ultrapure water. All measurements were conducted at room temperature.

### 2.5. Cell Culture

Raw 264.7 cells were cultured in high-glucose Dulbecco’s modified Eagle’s medium (DMEM; Hyclone, UT, USA), 10% fetal bovine serum (FBS; Gibco BRL, NE, USA), and 1% penicillin/streptomycin (Macgene, Beijing, China) at 37 °C in a humidified 5% CO_2_ atmosphere. When cells reached 80% confluence, 30 μg/mL of BMs were added and incubated for 24 h before conducting the follow-up experiments.

### 2.6. BMs Internalization into Magcrophages

To visualize the intracellular localization of BMs, sulfo-cyanine5 NHS ester (Abcam, ab146459, Cambrige, UK) was used to label BMs. Raw 264.7 cells were incubated with labeled BMs at 37 °C for 3 h. After incubation, Raw 264.7 cell membrane was stained with DIO membrane fluorochrome at 37 °C for 20 min (Beyotime Biotechnology, Shanghai, China), and the cell nucleus was stained with Hochest 33342 (Sigma, St. Louis, MO, USA) at 37 °C for 10 min, and then observed the images by laser confocal microscope (Leica SP8 STED 3X, Mannheim, Germany).

### 2.7. Immunocytofluorescence (ICF) Assay

To check the fate of BMs after entering the cell, Raw 264.7 cells were seeded in confocal dishes at 80% confluence and incubated with BMs (30 μg/mL) for the designated time. The cells were collected and confocal laser imaging was performed at 0 h, 3 h, 6 h, 12 h, 24 h, 48 h, 72 h, 96 h, and 120 h, 144 h, respectively. Specifically, the supernatant of cells was discarded and replaced with fresh medium at 6 h to remove extracellular BMs. at 0 h, 3 h, 6 h, 12 h, 24 h, 48 h, 72 h, 96 h, and 120 h, 144 h, respectively (the supernatant of cells was discarded and replaced with fresh medium at 6 h). 

After incubation, cells were washed with PBS (10 mM, pH 7.4) and fixed with 4% paraformaldehyde for 15 min, and then permeabilized with 0.5% Triton X-100 for 20 min and blocked with 10% bovine serum albumin (BSA) for 1 h at room temperature. Afterwards, cells were incubated with the anti-magnetosomes antibody overnight at 4 °C, then washed with PBS three times, and incubated with FITC goat anti-rabbit antibody (Beyotime Biotechnology, Shanghai, China) at room temperature for 1 h. Finally, cells were stained with DAPI (Macgene, Beijing, China) for 5 min and images were obtained by laser confocal microscope (Leica SP8 STED 3X, Mannheim, Germany).

### 2.8. Prussian Blue Staining

Raw 264.7 cells incubated with BMs were subjected to Prussian blue staining (Solarbio life sciences, Beijing, China) to evaluate the degradation of the inorganic core of BMs inside the cells. The cells were collected and Prussian blue staining were performed at 0 h, 3 h, 6 h, 12 h, 24 h, 48 h, 72 h, 96 h, and 120 h, 144 h, respectively (The culture medium was discarded and replaced with the fresh one at 6 h). Cells were first fixed with 4% paraformaldehyde for 15 min and stained with Prussian blue dye by incubating at 37 °C for 30 min, followed by washing with ultrapure water twice and stained with Prussian blue counterstain for 30 to 60 s. Finally, cells were washed with ultrapure water and imaged by EVOS microscope (Life Technologies, Carlsbad, CA, USA). 

### 2.9. Animal Experiment

All animal experiments were conducted in accordance with the institutional guidelines approved by the Institutional Ethical Committee of Animal Experimentation of the National Center for Nanoscience and Technology. Animals received care following the Guidance Suggestions for the Care and Use of Laboratory Animals. Healthy male 6–8week-old C57BL/6NCrl (C57) mice, SPF grade, were provided by Charles River Laboratories. Laboratory animal license number: Beijing SYXK 2017-0033 Beijing SYXK 2017-0022.

### 2.10. Mice Magnetic Resonance Imaging (MRI)

The 6–8 week-old male C57 mice were divided into three groups with three mice in each group for MRI. The clinical dosage of ferroferric oxide is around 7.5 μmol Fe/kg [33]. Sun et al. have estimated that LD50 of BMs is 62.7 mg/kg in rats [22]. Therefore, we decided to use half of the LD50 value as the high dosage and a low dosage that is 1/4 of the high one. Three groups of mice were intravenously injected with 100 μL PBS, 100 μL PBS containing BMs at 8 mg/kg (5 mg Fe/kg) and 32 mg/kg (20 mg Fe/kg), respectively. Then magnetic resonance imaging (MRI) was performed regularly at designated time-points to observe the signal changes of nuclear magnetic resonance in mice. The T2-weighted MR imaging of the BMs in mice was performed at room temperature with 7 T (BioSpec70/20USR, Bruker, Germany), using a circular polarized 1H mouse whole-body RF coil and corresponding animal bed. The experimental parameters were as follows: pulse sequence: T2-TurboRARE/Bruker: RARE, TE: 40 ms, TR: 3000 ms, TA: 01 (min): 15 (s), FA: 90°, Slice thickness: 1 mm, FOV: 40 × 40 cm^2^, ETL: 10, and MTX: 256 × 256.

### 2.11. Immunohistochemistry (IHC) and HE Staining of Mice Tissue

Haematoxylin and eosin (HE) staining and immunohistochemical (IHC) analysis were performed on paraffin-embedded sections of mice liver and spleen tissues. Tissues were first fixed by immersion in 4% paraformaldehyde, then embedded in paraffin. And Paraffin sections were dewaxed with xylene, gradient alcohol hydration. After dewaxing and hydration, endogenous peroxidase blocking was carried out (0.3% H_2_O_2_ for 25 min). Afterwards, the sample was incubated with F4/80 primary antibody, goat anti-mouse secondary antibody labeled with HRP, and finally stained with DAB. HE staining was performed following a standard protocol. The staining results were captured by microscope (Nikon E100, Tokyo, Japan).

### 2.12. The Distribution of BMs in Mice

The 6–8 week-old male C57 mice were divided into ten groups with three mice in each group for Prussian blue staining. Prussian blue staining was used to visualize the distribution of BMs in mouse tissue. The mice were intravenously injected 100 μL PBS containing 32 mg/kg BMs (20 mg Fe/kg). At the designated time point, three mice were sacrificed, and the liver, spleen, heart, lung, kidney, intestine, and brain from each were removed, fixed with paraformaldehyde, and embedded with paraffin for ultra-thin sectioning. The ultra-thin sections were stained with Prussian blue and images were acquired.

### 2.13. ICP-MS

The inductively coupled plasma-mass spectrometry (ICP-MS) analysis was performed with a Thermo ICAP-QC Series ICP-MS. The 6–8 week-old male C57 mice were divided into ten groups with three mice in each group for detecting Fe concentration. The mice were injected intravenously with magnetosome suspension at a dosage of 32 mg/kg (20 mg Fe/kg). Then, the liver, spleen, blood, urine, and feces samples were obtained at the designated time point. To prevent the blood remaining in the tissues from interfering with the determination of iron level, cardiac perfusion was performed before harvesting tissues. 

### 2.14. Cardiac Perfusion

Mice were anesthetized by injecting 200 μL 4% chloral hydrate into the abdominal cavity. The limbs of mice were fixed on the anatomical table. The xiphoid process was then lifted with tweezers, the chest was cut with scissors, and the pericardium was torn open with tweezers, exposing the heart. The right auricle was cut open with scissors, and the prepared saline syringe was inserted into the left ventricle to conduct the perfusion. The perfusion was ended when the fluid out of the right auricle was clarified.

### 2.15. Statistical Analysis

All the quantitative data in our studies were subjected to statistical analysis and were expressed as mean ± standard deviation (SD). The statistical analysis was performed with GraphPad Prism statistics software. *p* values of less than 0.05 were considered to be statistically significant. 

## 3. Results

### 3.1. The Characterization of the Magnetosomes (BMs)

**Figure 1:**
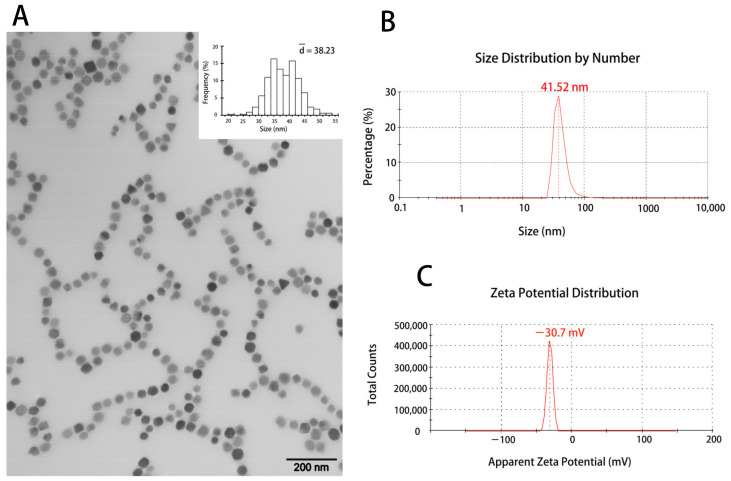
Characterization of bacterial magnetosomes (BMs). (**A**) The transmission electron microscopy image and the size distribution of BMs. The size distribution was calculated by Nano Measurer software based on the TEM results. Scale bars: 200 nm. (**B**) The size distribution of BMs determined by Zetasizer Nano ZS (PDI = 0.368). (**C**) The Zeta potential distribution of BMs determined by Zetasizer Nano ZS.

The BMs were purified from *Magnetospirillum gryphisiwaldense* MSR-1 magnetotactic bacteria by the ultrasonic crushing method [24]. The transmission electron microscopy (TEM) was used to visualize the morphological characteristics of BMs (Figure 1A). The average diameter of BMs was about 38 nm (Figure 1A). It can also be observed that purified BMs have good dispersibility and were organized in chains. Dynamic light scattering using Zetasizer Nano ZS confirmed the size distribution of the BMs (Figure 1B), and the zeta potential of the BMs was around −30.7 mV (Figure 1C). 

### 3.2. The Distribution of BMs in Mice by MRI

**Figure 2:**
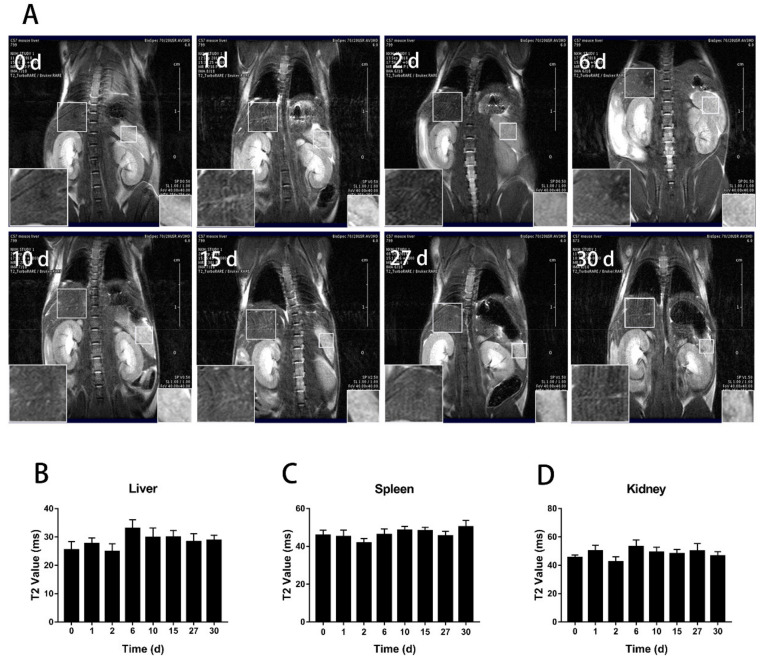
T2-weighted MR imaging (7.0 T) of mice after tail vein administration of PBS. (**A**) T2 weighted imaging at different time points after intravenous injection of PBS. The lower-left corner of each image is a partial enlargement of the mouse liver and the lower-right corner is the enlargement of the mouse spleen. Changes of the transverse relaxation time (T2) of the liver (**B**) the spleen (**C**) and the kidney (**D**) in mice at the designated time points were calculated.

**Figure 3:**
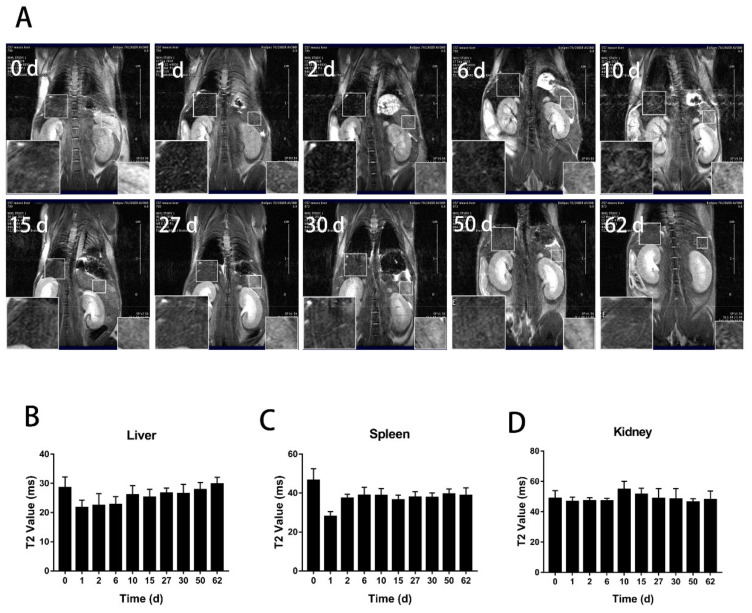
T2-weighted MR imaging (7.0 T) of mice after tail vein administration of BMs at the dosage of 8 mg/kg. (**A**) T2 weighted imaging at different time points after intravenous injection of BMs. The lower-left corner of each image is a partial enlargement of the mouse liver and the lower-right corner is the enlargement of the mouse spleen. Changes of the transverse relaxation time (T2) of the liver (**B**) the spleen (**C**) and the kidney (**D**) in mice at the designated time points were calculated.

**Figure 4:**
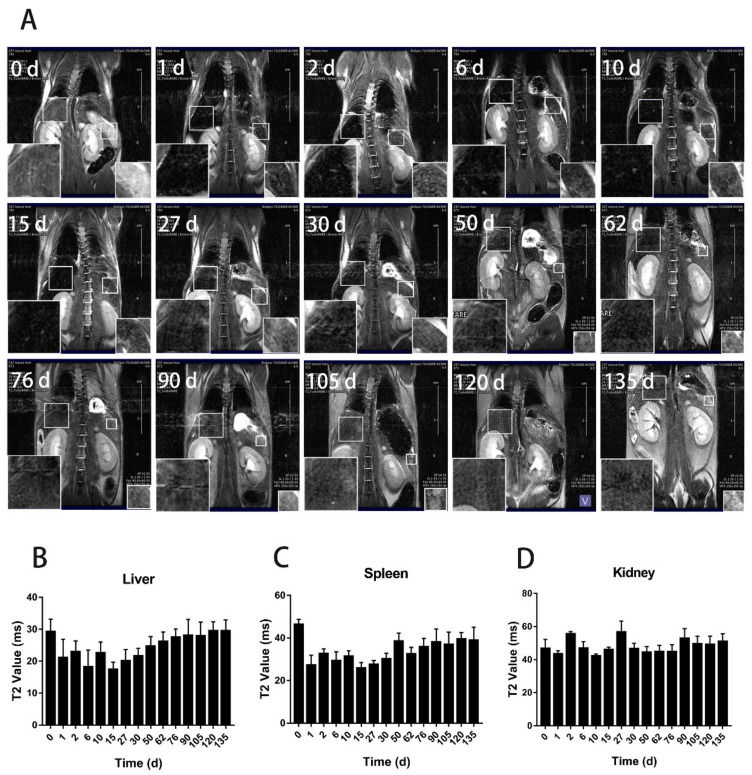
T2-weighted MR imaging (7.0 T) of mice after tail vein administration of BMs at the dosage of 32 mg/kg. (**A**) T2 weighted imaging at different time points after intravenous injection of BMs. The lower-left corner of each image is a partial enlargement of the mouse liver and the lower-right corner is the enlargement of the mouse spleen. Changes of the transverse relaxation time (T2) of the liver (**B**) the spleen (**C**) and the kidney (**D**) in mice at the designated time points were calculated.

To track the distribution and clearance of BMs after tail administration, magnetic resonance imaging (MRI) was performed on mice at the designated time points from day 0 to day 62 and 135 for the low and high dose, respectively, when the transverse relaxation time (T2) nearly returned to the control level (Figure 2). The result showed that for the low dosage group (8 mg/kg, BMs), there were significant changes in the liver and spleen on the first and second days after injection. The liver signal returned to the normal level on the tenth day (Figure 3), but the spleen became normal rather quickly on the sixth day. In the high dosage group (32 mg/kg, BMs), MRI signals in the liver and spleen of mice were also changed significantly from the first day. The transverse relaxation time (T2) was the shortest at approximately day 15, indicating that the amount of BMs was mostly accumulated in these two organs. After 120 days, the signal of the liver became normal, suggesting BMs were discharged from the liver. However, T2 of the spleen returned to the control level slightly earlier than the liver, which is at approximately 90 days (Figure 4). Noticeably, there were no significant changes in the kidney in all three groups, revealing that BMs do not accumulate in the kidney. The recovery of T2 further confirms that BMs were discharged from mice. 

### 3.3. The Tissue Ultrathin Section Staining

**Figure 5:**
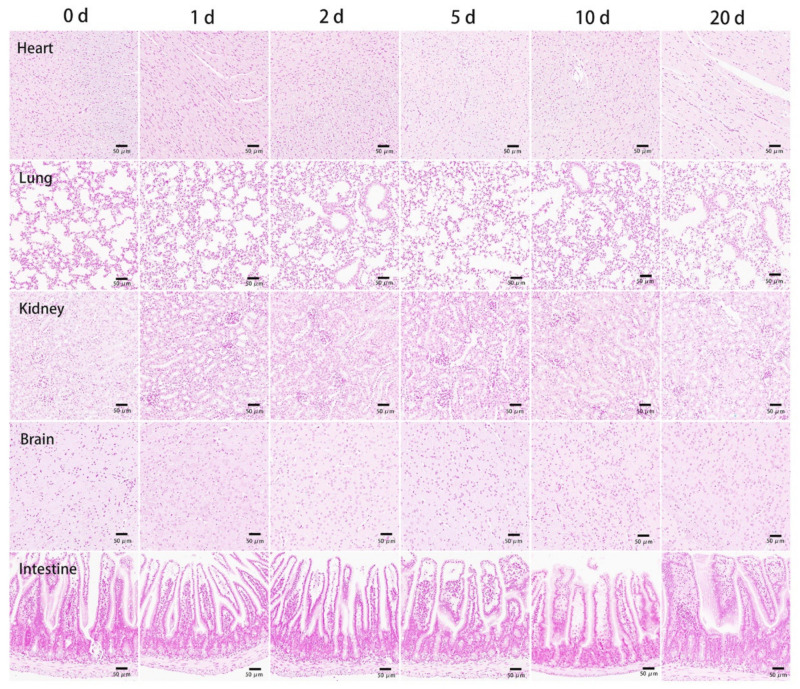
Prussian blue staining of the tissue ultrathin section of the heart, lung, kidney, brain, and intestine at the designated time points after tail vein injection of BMs at the dosage of 32 mg/kg. Scale bars: 50 μm.

**Figure 6:**
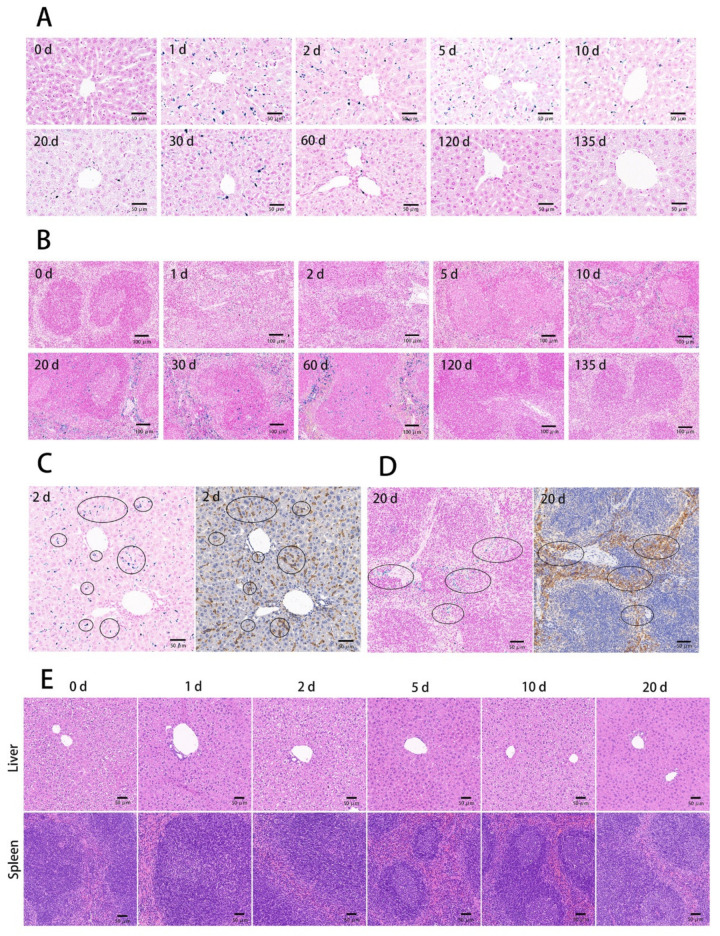
The liver and spleen ultrathin section staining at the designated time points after tail vein injection of BMs at the dosage of 32 mg/kg. Prussian blue staining images of the liver tissue (**A**) and the spleen tissue (**B**) were shown with scale bars of 50 μm. Comparisons between Prussian blue staining of the tissues and the immunohistochemical staining of macrophages for the liver second days (**C**) and spleen twentieth days (**D**) after BMs were injected intravenously and images were at scale bars of 50 μm. (**E**) HE staining of the liver and spleen tissue ultrathin sections at the designated time points after tail vein injection of BMs at 32 mg/kg. Scale bars: 50 μm.

To confirm BMs targeted organs and cells after being injected into the mice, we conducted Prussian blue staining on mice tissues after the continuous ultra-thin section. Briefly, at the designated time point, mice were sacrificed, and the heart, liver, spleen, lung, kidney, brain, and intestine were obtained for the ultrathin sectioning. Additionally, macrophages in the liver and spleen were subjected to immunohistochemical staining using F4/80 as the marker. The Prussian blue results showed that BMs were mainly accumulated in the liver and spleen after entering and circulating in the blood. No blue coloration was founded in other organs, indicating few BMs were accumulated at other parts of the body (Figure 5). BMs were distributed more evenly near the liver portal triad than in the spleen, where the images show deeper darkness in the parenchymal marginal area (Figure 6). Consistent with MRI results, Prussian blue staining also showed that the distributions of BMs in the organs decreased and cleared at 90 days with the high dosage injection. Additionally, the results of Prussian blue and immunohistochemical staining of the liver and spleen showed that the localization of BMs was the same as the location of macrophages, indicating the targeted cells of BMs after intravenous injection were macrophages in the liver and spleen (Figure 6C,D).

Next, hematoxylin-eosin (HE) staining was carried out to determine whether the BMs cause damage to the liver and spleen after tail vein injection into mice. No infiltration of inflammatory cells, deposition of foam cells, plaques, cell degeneration, and necrosis were founded (Figure 6E), indicating that BMs barely cause damage to the organs.

### 3.4. ICP-MS Analysis

Quantitative measurement of the iron element using inductively coupled plasma mass spectrometry (ICP-MS) was performed to detect changes of the iron level in the liver, spleen, blood, urine, and feces after injection of BMs at the dose of 32 mg/kg. Since the blood has a high level of iron, to reduce the interference of the blood in organs, cardiac perfusion was carried out to flush out the blood in the organs until the color of the liver changed from purple to khaki.

**Figure 7:**
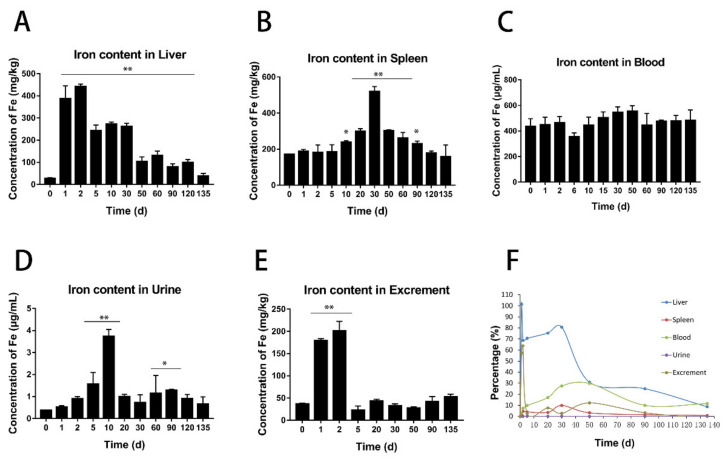
ICP-MS analysis of the iron content in various samples after injection of BMs at the dosage of 32 mg/kg. Changes of the iron content in the liver (**A**), spleen (**B**), blood (**C**), urine (**D**), and feces (**E**) and the proportion of total iron in these samples at the designated time points (**F**). (* *p* < 0.05, ** *p* < 0.01 means significantly different from pre-injection level.).

The results showed that the iron level in the liver increased sharply in a short time after intravenous injection of BMs, from 10–20 mg/kg to more than 400 mg/kg. The iron level in the liver was the highest in the first two days and began to decrease, but remained significantly high for 30 days, and returned to the base level gradually (Figure 7A). In the spleen, the iron level did not show noticeable change within the first 10 days, but increased significantly after 10 days, and reached the peak that was twice the base level at around day 30 (Figure 7B). The base iron level in the blood is high and has a slight increase at day 10 after injection and gradually returned to the pre-injection level at day 50 (Figure 7C). Compared to other tissues, the base level of the iron in the urine was less than 1 µg/mL [34] and the absolute change values are not large, even though there was a gradual increase from the first day and peaked on the 10th day with the amount at 4 µg/mL, which is 10 times more than the basal level (Figure 7D). It is also noticeable that the level lowered to a few times higher than the normal at day 20 and remained for 3–4 months. However, the increase of iron in feces was striking in the first two days, then returned to the normal level after two days (Figure 7E). Comparisons of changes of the percentages of iron occupying the total injected amount in different tissues (Figure 7F), we found in the first two days, a majority of injected BMs accumulated in the liver, and approximately 1/2 of the total amount were discharged to feces. We think this is probably because BMs were infiltrated to the bile ductule and further went to the duodenum with the secretion of bile. As time went on, BMs were degraded into small particles and iron ions, leading to an increase in blood and spleen. At the later stage, iron ions were slowly eliminated from the body through urination. The iron level in mice returned to close to the normal 3–4 months after the injection.

### 3.5. The Internalization and Elimination of BMs in Macrophages

The colocalization of BMs with macrophages in the liver and spleen suggested they entered macrophages, resulting in the accumulation in the liver, spleen, and blood that have a large number of macrophages. In the meantime, the iron level peak in the urine should be contributed by iron ions, suggesting BMs undergo degradation in the macrophages.

**Figure 8:**
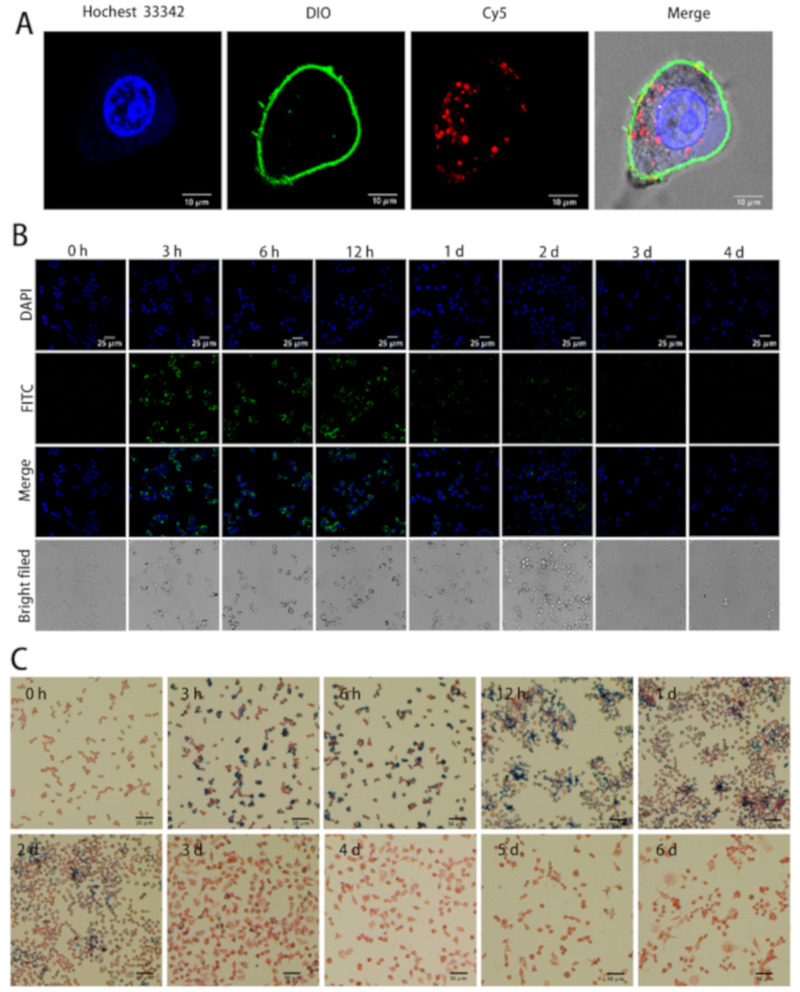
The cellular internalization and elimination of BMs in macrophages. (**A**) The inter-nalization of BMs in Raw 264.7 cells. Scale bars: 10 μm. Hochest 33342, DIO, and Cy5 were taken to stain cell nucleus, cell membrane and BMs into blue, green, and red respectively. (**B**) The laser confocal imaging results of Raw 264.7 cells incubated with BMs of 30 μg/mL at different time intervals. The blue shows the nuclei and the green refers to proteins on the BMs membrane. Scale bars: 25 μm. (**C**) The Prussian blue staining of Raw 264.7 cells incubated with BMs of 30 μg/mL at different time intervals. The red shows the cell nuclei, and the blue indicates the inorganic core of BMs. Scale bars: 50 μm.

To verify that BMs were internalized by macrophages, we performed the immunofluorescence (IF) assay to observe the localization of labeled BMs. As shown in Figure 8A, confocal analysis showed that BMs were ingested by Raw 264.7 cells and distributed between the membrane and the nucleus.

To analyze the degradation of magnetosomes in cells, we conducted immunocytofluorescence (ICF) experiments using antibodies against BMs to detect the membrane integrity of BMs at different time intervals after incubation of BMs with Raw 264.7. The inorganic iron core was also monitored using Prussian blue staining. Laser confocal imaging of ICF showed that green fluorescence signals enhanced apparently at 3 h after incubation with BMs, the intensity increased as the incubation continued and lasted until 12 h (Figure 8). The signal reduced significantly after 24 h and disappeared after 72 h. Two factors may explain the gradual signal fade-out: one is the magnetosome membrane proteins and lipids break-down in macrophages; the other is the proliferation of cells causing BMs to be divided into daughter cells which were therefore diluted. 

To check the degradation of BMs inorganic core in cells, we performed the cellular Prussian blue staining. As shown in Figure 8C, as cells divided, BMs were distributed among more cells in the first two days. The blue color disappeared significantly on day 3, indicating the inorganic BMs core was mostly broken down in the cell, and the degradation was almost complete within 4 days.

## 4. Discussion

In this study, we tracked the distribution and clearance of bacterial (MSR-1) magnetosomes in major organs for up to 135 days after intravenous injection into mice using MRI and various methods. MRI signal decay can be detected in the first two days for the low dosage and a few weeks for the high dosage. This is expected because almost half of BMs went to feces in the first two days (discussed below) and the particles uptaken by macrophages were mostly eliminated in 72 h. The consistency of MRI results and others confirms the high sensitivity of BMs as a contrast agent. ICP-MS measures both BMs and iron ions and is very sensitive; besides systemic measurement errors, the fact that BMs are not evenly distributed in the liver and spleen, variations of the individual mouse, and the slight difference of the excretion amount have caused variations of the quantitative ICP-MS results. Therefore, the averaged results only reflect trends of changes in different tissues and organs over time, which are consistent with MRI and Prussian blue staining results.

The clinical dosage of ferroferric oxide as an MRI contrast agent is approximately 7.5 μmol Fe/kg body weight [33]. Since BMs have been reported to have high biocompatibility and to enhance contrast of the targeted site, we have tried 8 and 32 mg/kg of BMs which are approximately 5 and 20 mg Fe/kg, corresponding to 10 and 50 times the clinical dosage. The tissue ultrathin section staining results showed that even at high dosages, few BMs were accumulated at the heart, lung, kidney, and brain. Besides, all tissues appear normal without any damages, including the liver and spleen, proving again that BMs have high biocompatibility. 

Our results are consistent with Sun et al. who reported BMs accumulated in the liver, but they did not find BMs present in other organs [22]. However, we found the spleen was another target organ and we think this can be explained by the presence of a lot of macrophages in the spleen. This is also confirmed by the observation of the Prussian blue staining (Figure 6D) that BMs mostly distributed in the marginal zone of the spleen parenchyma where macrophages dwell. The result also suggests that BMs can be an MRI contrast agent for imaging the liver and spleen. In the case when other targets than the liver and spleen are desired for the disease treatment or diagnosis, modifications of BMs should be performed to avoid macrophage uptake.

The clearance time of BMs in mice was positively correlated with the injection amount. When the amount was 8 mg/kg, the majority of BMs were eliminated from the liver at around 30 days after injection. However, at the dosage of 32 mg/kg, the clearance took 120 days. From the ICP-MS, we estimated that approximately 1/2 of the injection infiltrated the bile duct and went to the feces in the first two days, while the remaining BMs were degraded into smaller particles and eventually became iron ions and eliminated through urination [35]. The clearance from the body took more than 4 months with the iron level in the urine still a little higher than pre-injection. Several reasons could contribute to this long-term clearance: one is the degraded iron ions can be stored in the body iron reservoir system [36]; the other is that part of BMs can be re-absorbed by the intestine through enterohepatic circulation [37,38]. 

## 5. Conclusions

To summarize, we reported for the first time a long-term follow-up of the distribution and clearance of BMs in different tissues and organs in mice using a combination of several methods including MRI. Our study further confirmed that BMs as an MRI contrast agent have sufficient spatial resolution and high sensitivity. Meanwhile, we found the major targeting organs of BMs, the length of time, and two main paths for BMs to be cleared from mice. The targeting and biodegradability of the BMs suggest that it has the potential as a contrast agent for imaging liver and spleen, or other organs and tissues for disease treatment or diagnosis after modification to avoid macrophage uptake. The results may be useful in the clinical application and contribute to the biosafety assessment of BMs.

## Data Availability

The data presented in this study are available on request from the corresponding author.
